# Enhanced Antitumor Effect in Liver Cancer by Amino Acid Depletion-Induced Oxidative Stress

**DOI:** 10.3389/fonc.2021.758549

**Published:** 2021-11-02

**Authors:** Keiichiro Okuda, Atsushi Umemura, Seita Kataoka, Kota Yano, Aya Takahashi, Shinya Okishio, Hiroyoshi Taketani, Yuya Seko, Taichiro Nishikawa, Kanji Yamaguchi, Michihisa Moriguchi, Hayato Nakagawa, Yu Liu, Yasuhide Mitsumoto, Yoshihiro Kanbara, Toshihide Shima, Takeshi Okanoue, Yoshito Itoh

**Affiliations:** ^1^ Molecular Gastroenterology and Hepatology, Graduate School of Medical Science, Kyoto Prefectural University of Medicine, Kyoto, Japan; ^2^ Department of Gastroenterology, The University of Tokyo, Tokyo, Japan; ^3^ Department of Gastroenterology and Hepatology, Saiseikai Suita Hospital, Suita, Japan

**Keywords:** L-asparaginase, glutamine, asparagine, lenvatinib, liver cancer, glutamine synthetase, asparagine synthetase, oxidative stress

## Abstract

Hepatocellular carcinoma (HCC) is the most common type of primary liver cancer. HCC cells consume large amounts of glutamine to survive, but can adapt to glutamine depletion in the presence of an exogenous asparagine. L-asparaginase (ASNase) converts glutamine and asparagine to glutamate and aspartate, respectively, and has been used to treat leukemia. Here we examined the effects of ASNase treatment on HCC cells and explored the potential impact of combining ASNase with the tyrosine kinase inhibitor lenvatinib (Len) for HCC treatment. Cell viability and death of HCC cell lines treated with either Len or ASNase alone or with Len and ASNase combined were determined. We assessed mRNA and protein expression levels of glutamine synthetase (GS) and asparagine synthetase (ASNS) by real-time quantitative PCR and immunoblotting. The antitumor effect of the combination therapy relative to Len or ASNase monotherapy was also evaluated in a xenograft tumor mouse model. ASNase treatment inhibited growth of SNU387 and SNU398 HCC cells, which have low GS and high ASNS expression levels, respectively, but did not clearly inhibit growth of the other cell lines. Len plus ASNase combination therapy synergistically inhibited proliferation and induced oxidative stress leading to cell death of some HCC cells lines. However, cell death of Huh7 cells, which express ASCT2, an important glutamine transporter for cancer cells, was not affected by the combination treatment. In a xenograft model, Len combined with ASNase significantly attenuated tumor development relative to mice treated with Len or ASNase alone. ASNase-mediated targeting of two amino acids, glutamine and asparagine, which are indispensable for HCC survival, induces oxidative stress and can be a novel cancer treatment option that exerts a synergistic effect when used in combination with Len.

## Introduction

Hepatocellular carcinoma (HCC) is the sixth most common malignant tumor and the fourth leading cause of cancer-related death worldwide (1). Many HCC patients are diagnosed at advanced stages of disease, and thus local treatment options, including curative hepatic resection, tumor ablation, or transarterial therapy, are not suitable. Therefore, systemic treatments for advanced HCC are urgently needed. For systemic treatment of HCC, tyrosine kinase inhibitors (TKIs) such as sorafenib, regorafenib, and lenvatinib (Len) are widely used. Although the efficacy of TKIs is largely insufficient, Len did exhibit a significantly higher response rate relative to sorafenib as a first-line therapeutic option for advanced HCC in the phase III REFLECT trial (2).

Recent studies revealed that tumor cells, including HCC cells, take advantage of metabolic alterations to advance proliferation and survival (3). Tumor cells have increased demand for amino acids and the activity of amino acid metabolic pathways is thought to be altered in these cells (4). Previous studies convincingly demonstrated that glutamine is consumed by proliferating tumor cells with preference compared to other amino acids. Moreover, glutamine levels are substantially lower in the tumor core relative to peripheral regions (5).

Importantly, exogenous asparagine becomes an essential amino acid for cancer cells and can maintain protein synthesis as extracellular glutamine levels decline near the cells (6) in addition to promoting growth and survival of glutamine-deprived tumor cells. Asparagine-mediated rescue of tumor cell proliferation requires glutamine synthetase (GS), suggesting that glutamine supplied by *de novo* synthesis compensates for extracellular glutamine depletion. GS expression is reported to increase gradually with the development of liver carcinogenesis to achieve high expression levels in human HCC (7). In addition, high GS expression levels have been associated with poor prognosis in patients with HCC. *In vitro* studies indicated that GS influences HCC cell migration by mediating the epithelial-mesenchymal transition (7).

Recent advances in the understanding of amino acid metabolism revealed that targeting amino acids, especially glutamine and asparagine, in cancer therapy can be a promising strategy for the development of novel therapeutic agents. In fact, tumor cells that are exposed to glutamine-deficient conditions lose the ability to survive, proliferate, and metastasize (8). Glutamine and asparagine dependency is frequently altered in cancers, including HCC, and glutamine in particular is indispensable for development of HCC (9).

Although the mechanistic details are not fully clarified, L-asparaginase (ASNase), which converts glutamine and asparagine to glutamate and aspartate, respectively, has long been targeted for treatment of acute lymphoblastic leukemia (10). An examination of the antitumor mechanism of ASNase revealed that it also possesses glutaminase activity that depletes glutamine levels and plays an important role in induction of cell death. Glutamine has the highest structural similarity to asparagine relative to other amino acids (11), and ASNase suppressed both the growth and survival of glutamine-depleted cells as well as tumor growth. Clinical trials to assess the effects of ASNase in patients with cancer, including those with pancreatic cancer, are ongoing (12, 13). Consistent with these rationales, ASNase-mediated glutamine depletion is an effective treatment in a mouse model of HCC (14, 15). However, the activity of ASNase in combination with other anticancer drugs such as TKIs has not yet been explored.

In the present study, we considered a new approach to HCC combination therapy based on the co-administration of a commonly used TKI, Len, with ASNase. We explored the association between the effect of ASNase and expression levels of GS and asparagine synthetase (ASNS) in several HCC cell lines. We also demonstrate that ASNase yielded synergistic antitumor effects when used in combination with Len *in vitro* and *in vivo*, and confirmed that this combination therapy exerted growth-inhibitory and cell death-induction effects in HCC cells that were mediated through production of oxidative stress.

## Materials and Methods

### Human Liver Cancer Cells and Mouse Hepatocytes

The liver cancer cell lines Hep3B (RRID: CVCL_0326), SNU387 (CVCL_0250), SNU398 (CVCL_0077), and HepG2 (CVCL_0027) were obtained from ATCC. The mouse hepatocyte cell line AML12 (CVCL_0140) was also obtained from ATCC. Huh6 (CVCL_1296) and Huh7 (CVCL_0336) cells were purchased from the Japanese Collection of Research Bioresources Cell Bank (JCRB). All cells were obtained directly from cell banks that perform cell line characterizations and were passaged in our laboratory for fewer than 6 months after receipt. The cells were maintained according to instructions provided by the ATCC and JCRB and incubated with Len (CAS No.: 417716-92-8, ChemScene LLC, NJ, USA) and/or ASNase (ENZ-287, Prospec, NJ, USA) using the indicated concentration and time course. Relative cell viability was determined using a WST-8 method with Cell Count Reagent SF (#07553-15, Nacalai Tesque, Kyoto, Japan). Combination Index (CI) and dose-effect analyses were calculated using CompuSyn software (ComboSyn, Inc., Paramus, NJ, 07652 USA) according to the Chou-Talalay method (16). CI values were plotted against the fraction of affected cells (Fa) to represent the percentage of growth inhibition (CI-Fa plot). A CI value <1.0 indicates synergism of the combination. A lactate dehydrogenase (LDH) cytotoxicity assay was carried out using a Cytotoxicity LDH Assay Kit (#CK12, Dojindo Laboratories) according to the manufacturer’s instructions. Cells were incubated with indicated concentrations of drugs for 72 hours. Cytotoxicity was calculated as a percentage of the ratio of LDH release compared to controls having high levels of LDH.

### RNA Isolation and Real-Time Quantitative PCR

RNA was extracted using TRIzol (#15596018, Invitrogen/Thermo Fisher Scientific, MA) and further purified using chloroform and isopropanol. RNA (1 μg) was used to generate cDNA with the PrimerScript RT cDNA Synthesis Kit (#RR036A, Takara Bio, Shiga, Japan). The expression of individual genes was quantified by real-time qPCR using SYBR FAST qPCR Master Mix (#KK4602, KAPA BIOSYSTEMS, MA) and a LightCycler 96 Real-Time PCR system (Roche Diagnostics, Mannheim, Germany). Expression levels were normalized to the housekeeping control gene *GAPDH*. Primers used for real-time qPCR analyses are listed in Supplementary Table 1.

### Immunoblot Analysis

Harvested liver cancer cells were homogenized in RIPA buffer and equal amounts of liver homogenates were fractionated by SDS-PAGE and transferred onto a PVDF membrane. The membranes were incubated with antibodies to GS (#G2781, Sigma-Aldrich, MO), ASNS (#14681-1-AP, Proteintech, IL), ASCT2 (#8057), p70S6K (#9202), phospho-p70S6K (Thr389, #9205), S6 (#2217), phospho-S6 (#4858), 4EBP1 (#9452), phospho-4EBP1 (#2855), PARP (#9532) (from Cell Signaling Technology, MA), and β-actin (Sigma-Aldrich, MO).

### Immunofluorescence Analysis

A LIVE/DEAD Cell Imaging Kit (#R37601, Invitrogen) and ROS (reactive oxygen species) Assay Kit-Highly Sensitive DCFH-DA kit (#R252; DOJINDO Laboratories. Inc., Kumamoto, Japan) were used according to the manufacturer’s instructions for the immunofluorescence analysis of liver cancer cell lines, including Huh6 cells, treated with Len, ASNase, or Len plus ASNase. Briefly, cells were incubated in a 96-well plate with the indicated treatment for 72 h, followed by incubation with the LIVE/DEAD reagent for 15 min at room temperature, or DCFH-DA solution for 30min at 37°C. A BZ-X800 Fluorescence Microscope (Keyence corporation, Osaka, Japan) was used to observe live or dead cells and to detect ROS.

### Animals

The animal study was performed in accordance with Kyoto Prefectural University of Medicine (KPUM) guidelines for the care and use of live animals and was approved by the KPUM Institutional Animal Care and Use Committee (M2019-559). The mice used in this study for the xenograft model were female BALB/c nude mice aged 6-8 weeks and C57/BL6J male mice aged 20 weeks for determination of pharmacological impacts of ASNase on mature mice (Charles River Laboratories, Japan). The animals were maintained at KPUM in filter-topped cages with a 12 h dark/light cycle and given an autoclaved diet and water. To generate a xenograft tumor model, the flanks of 40 mice were inoculated with liver cancer cells (2.5 × 10^6^ cells) suspended in 100 μl PBS. Two weeks after the inoculation, mice having a tumor ≥3 mm were randomly divided into four groups: Control, Len, ASNase, and Len plus ASNase groups (n = 7–9 per each group). Len was administered orally daily (10 mg/kg in 3 mM HCl DMSO), while ASNase was injected intraperitoneally three times per week (5 U/g). The control group received saline instead of Len or ASNase. After 2 weeks of treatment, all the mice were sacrificed and analyzed. The relative tumor volumes were evaluated by comparing the final and initial volumes. Tumor volumes were calculated using the formula V = (L x W x W)/2, where V is tumor volume, W is tumor width, L is tumor length.

### Statistical Analysis

Data are presented as the mean ± standard deviation (SD) or the median with interquartile range, as indicated. For *in vitro* analysis, representative data from at least three independent experiments were shown. For the DEAD/LIVE and ROS stainings (**Figures 3** and **5**), 8-10 field images were captured and analyzed. Differences in means and two categorical variables were analyzed by student’s *t*-test using JMP8.0 (SAS Institute Inc., NC). Multiple comparisons were analyzed by Turkey’s multiple comparisons test (GraphPad Prism 6, GraphPad Software, CA). Significance was set at *P* values < 0.05 (not significant (N.S.), *P* > 0.05; *, *P* < 0.05; **, *P* < 0.01).

## Results

### Effects of Len and ASNase Treatment on Liver Cancer Cell Viability

We first examined the effect of Len, a TKI that is commonly used for HCC treatment, and ASNase on the survival of six liver cancer cell lines carrying specific mutations. Huh6, SNU398, and HepG2 cells harbor β-catenin mutations, while Hep3B and Huh7 cells have Fibroblast Growth Factor 19 (FGF19) mutations. SNU387 cells do not possess β-catenin or FGF19 mutations (Huh6: β-catenin+, FGF19−; Huh7: β-catenin−, FGF19+; SNU387: β-catenin−, FGF19−; SNU398: β-catenin+, FGF19−; Hep3B: β-catenin−, FGF19+; and HepG2: β-catenin+, FGF19−) (17). Notably, GS is a target of β-catenin (18), whereas FGF19 expression was reported to be associated with the clinical efficacy of Len (19).

**Figure 1:**
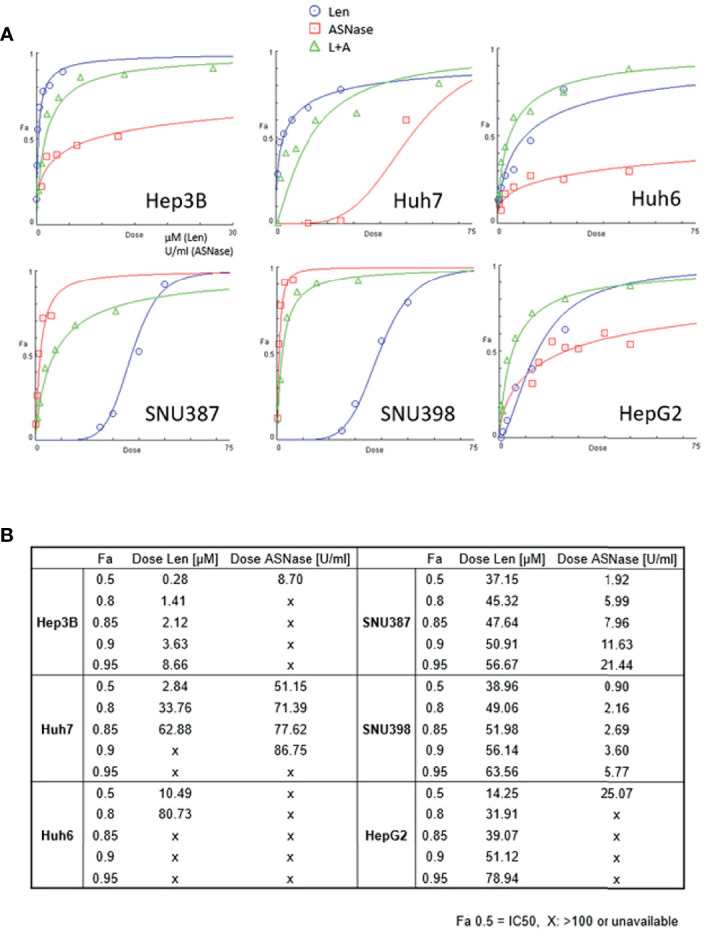
Effects of lenvatinib and L-asparaginase on liver cancer cell viability. **(A)** Dose-effect curves for the indicated cell lines were generated using cell viability data from WST-8 assays. The fraction of affected cells (Fa) representing the percentage of growth inhibition and dose-response plots are indicated for monotherapy and combination therapy (Len, blue; ASNase, red; Len plus ASNase, green). Drug doses that yield Fa 0.5 are required for a 50% inhibitory effect (equivalent to the IC50). **(B)** Summary of drug concentrations that inhibited cell survival by 50%, 80%, 85%, 90%, and 95%, indicated by Fa (Len, lenvatinib; ASNase, L-asparaginase). “X”, dose >100 or unavailable. Representative data from three independent experiments were shown.

Cell viability was measured using the WST-8 method after 72 hours of incubation with the indicated treatment. Len (**Figure 1A**, blue lines) induced dose-dependent growth inhibition in the four cell lines. In particular, Hep3B and Huh7 cells, which harbor an FGF19 mutation, showed maximal inhibition (Fa 0.5: 0.28 μM for Hep3B and 2.84 μM for Huh7 cells, where Fa 0.5 is equivalent to the half-maximal inhibitory concentration (IC_50_), which causes a 50% inhibition of the desired activity; **Figure 1B**). Huh6 and HepG2 cells exhibited a moderate degree of growth inhibition (Fa 0.5: 10.49 and 14.25 μM, respectively; **Figure 1B**). SNU387 and SNU398 cells were more resistant to Len than the other cell lines (Fa 0.5: 37.15 and 38.96 μM, respectively; **Figure 1B**). Specifically, ASNase (**Figure 1A**, red lines) caused a clear dose-dependent inhibition of the growth of SNU387 and SNU398 cells (Fa 0.5: 1.92 and 0.90 U/mL, respectively; **Figure 1B**), whereas Huh7 and HepG2 cells were partially resistant to this treatment (Fa 0.5: 51.15 and 25.07 U/mL, respectively). Notably, Huh6 cells did not reach 50% growth inhibition (Fa 0.5: > 100 U/mL).

We also confirmed that ASNase indeed depleted asparagine and glutamine concentrations but increased aspartate and glutamate levels in the cell culture media (**Supplementary Figure 1A**).

### The Combination of ASNase and Len Exerted Synergistic Effects on Liver Cancer Cells

**Figure 2:**
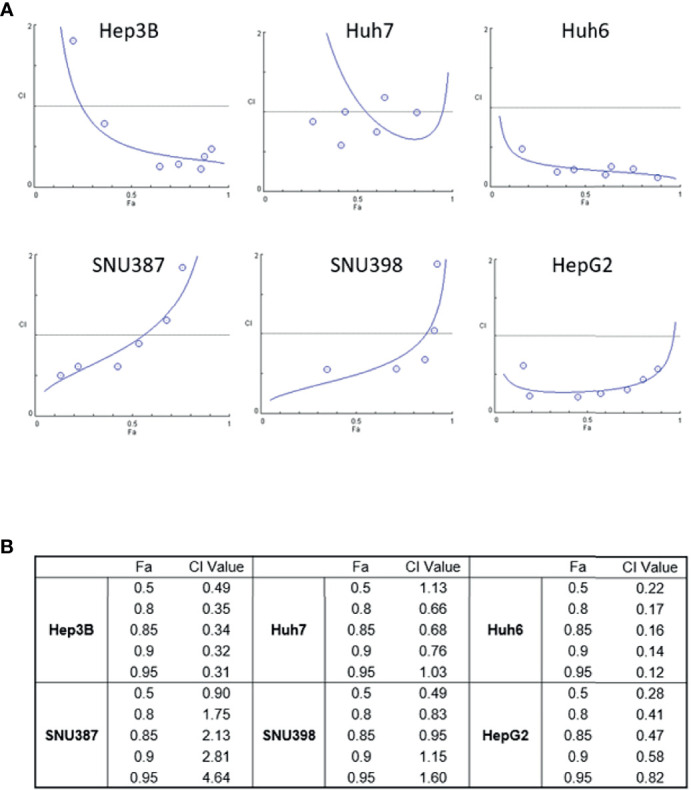
Synergistic effects of lenvatinib and L-asparaginase combination therapy on liver cancer cell viability. **(A)** Combination index (CI) showing the synergistic effect of lenvatinib (0-50 μM) and L-asparaginase (0–50 U/ml) on HCC cells. The CI value was determined using the CompuSyn algorithm and was plotted against Fa, which represents the percentage of growth inhibition (CI-Fa plot). A CI value <1.0 indicates synergism of the combination. **(B)** Summary of Fa 0.5, 0.8, 0.85, 0.9, 0.95 values and corresponding CI values. The strongest synergistic effect of lenvatinib and L-asparaginase combination therapy was seen with Huh6 cells. Representative data from three independent experiments were shown.

To explore the possibility that treatment with ASNase combined with Len inhibits liver cancer cell growth to a greater extent than either drug alone, we treated liver cancer cells with ASNase and Len combined. The results from WST-8 analyses were used to determine the Combination Index (CI), which is the fraction of affected cells (Fa) (**Figure 2**). A synergistic effect indicated by a CI value <1 for Len (0–50 μM) with ASNase (0–50 U/mL) was seen for all HCC cells tested (Fa 0.5), except for Huh7 cells (CI = 1.13). Surprisingly, the Len plus ASNase combination therapy had the highest synergistic effect on Huh6 cells, even though treatment of these cells with ASNase monotherapy did not reach Fa 0.5 (**Figures 1A, B**).

**Figure 3:**
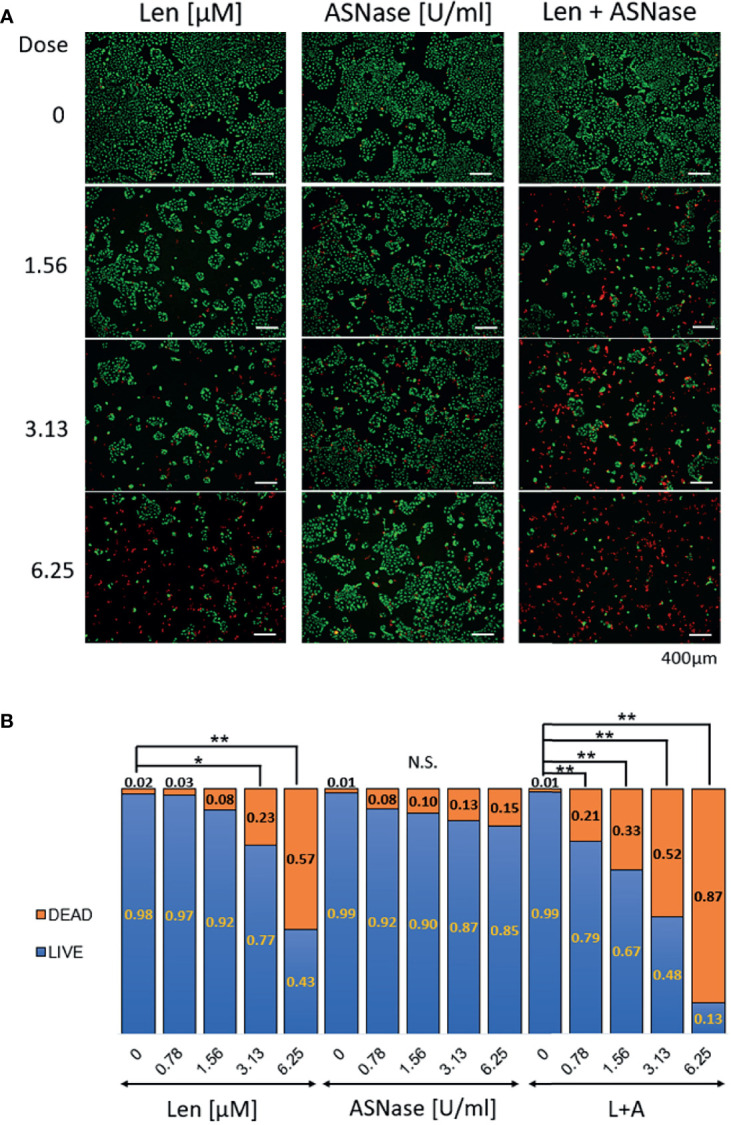
LIVE/DEAD staining of liver cancer cells treated with lenvatinib and L-asparaginase. Huh6 cells were treated with lenvatinib (Len, 0 – 6.25 μM) or L-asparaginase (ASNase, 0 – 6.25 U/ml) alone or in combination for 72 h and were then labeled with LIVE-Green and DEAD-Red probes. **(A)** Huh6 cells were treated with the indicated drugs, then stained with the fluorescence probes. Live cells show an intense and uniform green fluorescence, whereas dead or dying cells exhibit a predominantly red nuclear fluorescence. The samples were examined using fluorescence microscopy (BX-800, Keyence, Osaka, Japan). Scale bars, 400 μm. **(B)** Ratio of LIVE/DEAD cells after treatment with lenvatinib or L-asparaginase alone or in combination. Live or dead cells were quantified 72 h after incubation with the indicated drugs. All graphs represent the mean ± SD [Turkey’s multiple comparisons test, not significant (N.S.), *P < 0.05, **P < 0.01 *vs*. no treatment (0)]. For the DEAD/LIVE staining, 8-10 field images were captured and analyzed.

To confirm whether the combination of Len and ASNase induced cell death, LIVE/DEAD staining was performed on Huh6 cells (**Figure 3A**). The live cells showed intense and uniform green fluorescence, while the dead or dying cells exhibited a predominantly red nuclear fluorescence. In accordance with the WST-8 results, Len treatment had a moderate antitumor effect, whereas ASNase did not induce death of Huh6 cells. Importantly, the Len and ASNase combination treatment induced cell death to a much greater extent than the Len monotherapy (**Figure 3B**). These results are comparable with those of WST-8 analyses.

We also performed WST-8 assay for AML12, a normal hepatocyte cell line, and found that high dose of Len or ASNase, approximately 16 μM or 15 U/ml respectively, reduced cell proliferation, however, no synergistic effect indicated by a CI value <1 for Len (0–50 μM) with ASNase (0–50 U/mL) was achieved unlike in the case of HCC cell lines (**Supplementary Figures 2A–C**). Taken together, these results suggest that ASNase exerts synergistic antitumor effects in concert with Len on liver cancer cells and is particularly effective on Huh6 cells.

### ASNase Is Effective in Liver Cancer Cells Having High ASNS and Low GS Expression Levels

**Figure 4:**
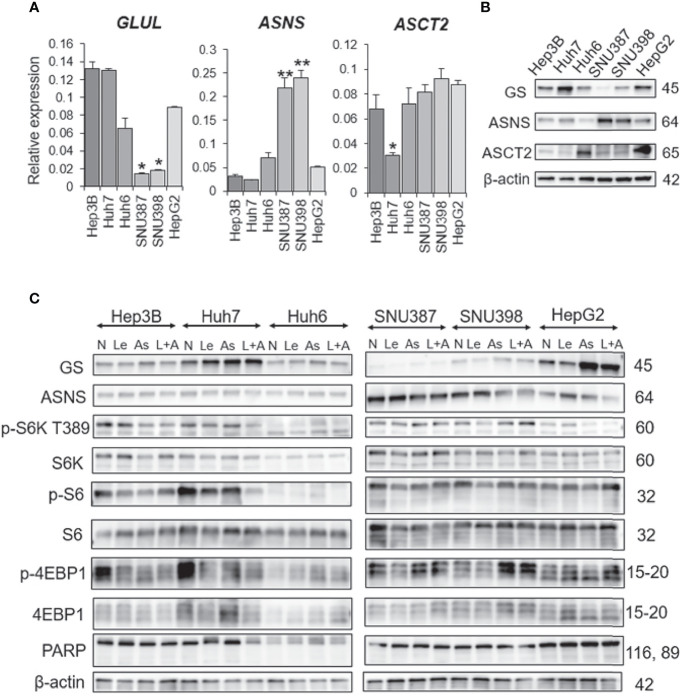
GS and ASNS expression determines L-asparaginase efficacy in liver cancer cells. **(A)** Relative expression of the *GLUL*, *ASNS*, and *ASCT2* mRNA in human HCC cell lines. All graphs represent the mean ± SD (N = 3 in each group, Turkey’s multiple comparisons test, *P < 0.05, **P < 0.01 *vs*. Hep3B cells). Expression levels were normalized to the housekeeping control gene GAPDH. **(B, C)** Immunoblot analysis of HCC cell extracts. Expression of the GS, ASNS, ASCT2, phospho-S6K(T389), phospho-S6, phospho-4EBP1, S6K, S6, 4EBP1, and PARP proteins was examined. β-actin was used as a loading control (N, no treatment; Le, lenvatinib; As, L-asparaginase; L+A, lenvatinib plus L-asparaginase). Representative data from three independent experiments were shown.

To investigate the mechanism by which ASNase reduces cell viability and induces cell death of liver cancer cells, we examined expression levels of GS and ASNS. Specifically, we analyzed messenger RNA (mRNA) expression levels of glutamate-ammonia ligase (*GLUL*), which encodes GS, and *ASNS* genes by real-time quantitative PCR (qPCR). SNU387 and SNU398 cells, which are sensitive to ASNase, showed lower GS expression than other cells, in addition, they also expressed higher ASNS levels than other cells at both the mRNA and protein level (**Figures 4A, B**). The amino acid contents in the culture media were analyzed and indicated in supplementary **Figure S2D**. The results suggest that SNU387 and SNU 398 consumed more extracellular glutamine (GLN) than other cells. Because of low GS expression, these two cell lines were more sensitive to asparagine (ASN) and GLN depletion by ASNase. The culture media from SNU387 and SNU 398 contained more ASN than other cells, which is consistent with higher ASNS expression. It is known that ASNS catalyzes the synthesis of asparagine (ASN) using glutamine (GLN). It may also lead to less GLN detected in the culture media from SNU387 and SNU 398 than others.

Interestingly, ASNase induced GS expression in cells with high GS expression (**Figure 4C**). Although ASNase alone was not effective on Hep3B, Huh6, or HepG2 cells, the combination of ASNase with Len synergistically suppressed viability of these cell lines.

We also examined the effects of ASNase on SNU387 cells with ASNS knockdown and Huh6 cells with GS knockdown. As expected, GS knockdown additively reduced the viability of Huh6 cells but not affect that of SNU387 cells in which GS expression was low. ASNS knockdown did not reduce the viability of both cell lines. The knockdown efficiency was confirmed with immunoblotting (**Supplementary Figures 3**, **4**). Next, cross-silencing experiments of GS in SNU387 and ASNS in Huh6 were performed (**Supplementary Figure 4**). These silencing did not affect the cell viability.

As Huh7 cells alone were resistant to the combination of ASNase and Len, we compared the expression levels of amino acid transporters in all cell lines. Among the selected transporters, expression of ASCT2 (*SLC1A5*), which is important for maintaining glutamine levels in tumor cells (20), was downregulated at both the mRNA and protein level in Huh7 cells (**Figures 4A, B**). In addition, Huh7 cells had the highest GS expression among all the liver cancer cell lines tested (**Figure 4B**). These results suggest that Huh7 cells do not depend upon extracellular glutamine for survival, but rather *de novo* glutamine synthesis or other metabolic alterations that provide energy sources.

### β-Catenin Mutation and mTORC1 Are Not Related to ASNase Efficacy

Huh6, SNU398, and HepG2 cells harbor β-catenin mutations, and GS is a β-catenin target. Unexpectedly, we found that the presence of a β-catenin mutation did not correlate with GS expression level or sensitivity to ASNase (**Figure 4B**). Therefore, we further investigated the synergistic effects of ASNase plus Len as a combination therapy. A previous study reported that the inhibition of glutamine-dependent mechanistic/mammalian target of rapamycin complex 1 (mTORC1) activation ameliorates liver cancer development driven by β-catenin mutations (18). We and another group previously reported that mTORC1 activation is sufficient for the spontaneous development of HCC in a mouse model in which the tumor suppressors tuberous sclerosis complex (TSC) 1 or TSC2 are lost (21, 22). Notably, mTORC1 activation is highly prevalent in cancer, including in 45% to 50% of HCC cases (23). Therefore, we evaluated mTORC1 activation by measuring phosphor-p70 ribosomal S6 kinase (p-S6K), phospho-S6 ribosomal protein (p-S6), and phospho-eukaryotic translation initiation factor 4E-binding protein 1 (p-4EBP1) expression levels in cell lines treated with the various agents. We found that basal mTORC1 activity was relatively high in Huh7 and Hep3B cells and Len or ASNase monotherapy reduced its activity, however, the combination therapy showed no differences compared to the monotherapies. Although the combination therapy reduced mTORC1 activity in Huh7 cells, none of the treatments yielded significant mTORC1 activation or suppression in Huh6 cells. Therefore, mTORC1 may not be a critical determinant of the sensitivity of Len plus ASNase combination therapy.

We evaluated apoptotic cell death using poly (ADP-ribose) polymerase (PARP) as an apoptotic marker (**Figure 4C**). ASNase did not regulate PARP levels either with or without Len co-administration in Huh6 cells. Next, we focused on reactive oxygen species (ROS) production.

### ASNase Induces ROS Production in Liver Cancer Cells

**Figure 5:**
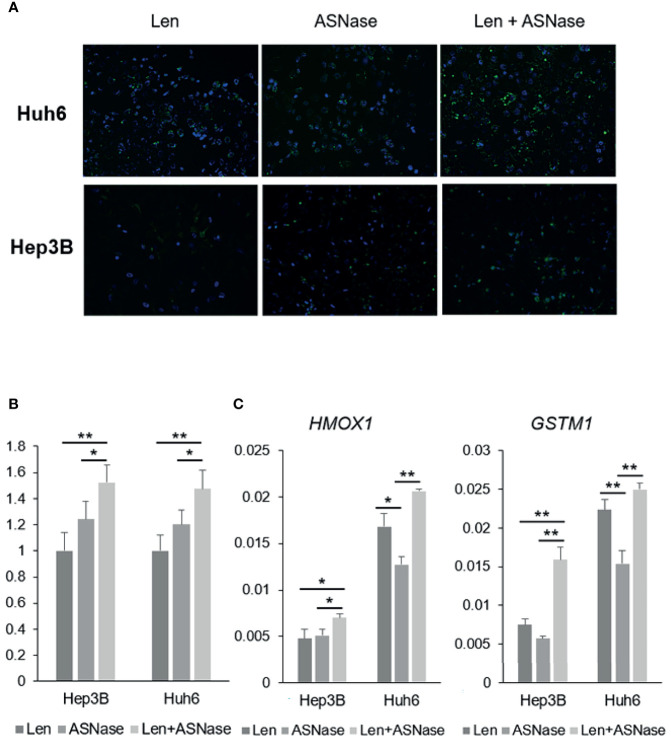
ASNase-induced ROS production. ROS staining of Hep3B and Huh6 liver cancer cells treated with lenvatinib (Len) or L-asparaginase (ASNase) alone or with Len + ASNase combination. HCC cells were incubated with indicated concentrations of the drugs for 72 hours. **(A)** ROS (reactive oxygen species) were stained with DCFH-DA probes. **(B)** Graphs represent ROS production normalized to Len treatment. For the ROS staining, 8-10 field images were captured and analyzed. **(C)** Relative expression of the GLUL, ASNS, and ASCT2 mRNA in human HCC cell lines. All graphs represent the mean ± SD (N = 3 per each group, expression levels were normalized to the housekeeping control gene GAPDH.) Turkey’s multiple comparisons test was used to evaluate the significance of differences (*P < 0.05, **P < 0.01).

Interestingly, glutamine-depleted tumor cells are reported to be vulnerable to vitamin C treatment due to a significant increase in ROS production (24). To explore the effect of ASNase on liver cancer cells, we performed a reactive oxygen species (ROS) assay. Len treatment was associated with ROS production in Hep3B and Huh6 cells, but ASNase treatment induced more ROS production than did Len treatment (**Figures 5A, B**). Notably, the combination of Len and ASNase demonstrated substantially more ROS production than ASNase monotherapy. We also analyzed some anti-oxidant genes by real-time qPCR. Among them, *HMOX1* and *GSTM1* were upregulated by the combination treatment and indicated in **Figure 5C**. These results suggest that ASNase induces oxidative stress in HCC cells, and Len and ASNase combination therapy enhances this stress response that contributes to the antitumor effect. Oxidative stress may thus play a role in the anticancer effects of ASNase.

### ASNase Sensitizes Liver Cancer Cells to Len in Nude Mice

**Figure 6:**
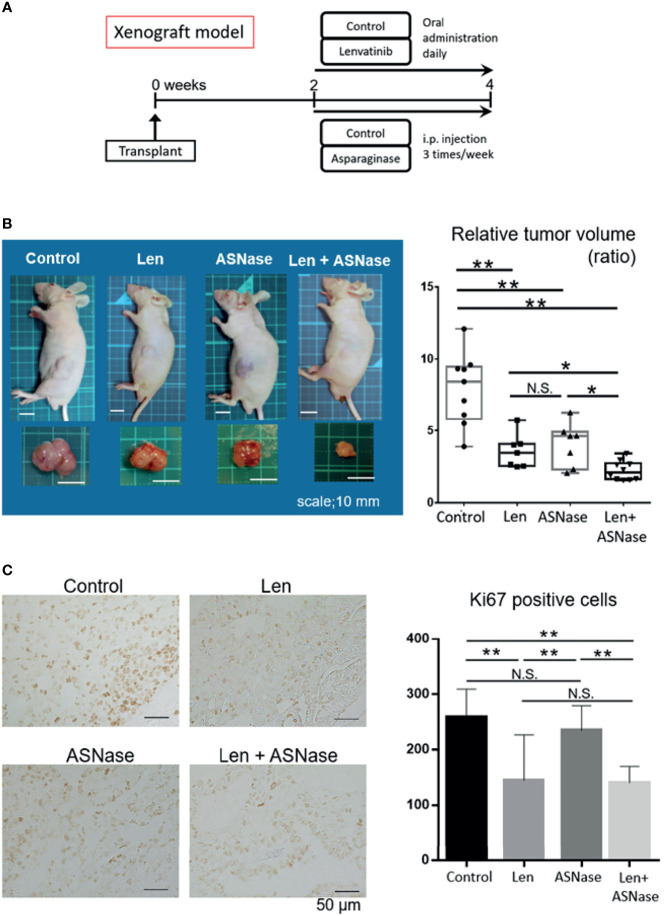
L-asparaginase sensitizes liver cancer cells to Lenvatinib in nude mice. BALB/c nude mice were inoculated with Huh6 cells, for which the combination therapy was most effective in *in vitro* assays. When tumors were palpable (≥3 mm) at day 14, the mice were treated with lenvatinib (Len), L-asparaginase (ASNase), combined with ASNase (Len + ASNase), or the vehicle control (Control) for an additional 14 days (n = 7–9 per group). **(B)** Macroscopic photos of inoculated mice and gross morphology of tumors at time of sacrifice. Squares on the photo background indicate 10 × 10 mm area (left). Relative tumor volume of the treated mice (right). Boxes in the graph indicate the median with interquartile range, and whiskers extend to the smallest and largest value. **(C)** Ki67 staining of xenografted tumors treated with the indicated drugs (left). The number of Ki67-positive cancer cells in the xenograft tumors is shown (right). The graphs represent the mean ± SD. (Turkey’s multiple comparisons test, not significant (N.S.), *P < 0.05, **P < 0.01).

Next, we tested the effects of the ASNase combined with Len in an *in vivo* mouse model. As a preliminary experiment, we injected ASNase intraperitoneally into wild-type mice once a day for eight consecutive days, and then examined the blood amino acid profiles. As expected, these mice exhibited asparagine and glutamine depletion, whereas aspartate and glutamate concentrations were increased (**Supplementary Figure 1B**). Next, we inoculated Huh6 cells, in which the combination therapy was the most effective, into the flanks of BALB/c nude mice. When tumors were palpable (≥3 mm) at day 14, the mice were randomly divided into four treatment groups: Len, ASNase, Len combined with ASNase (Len + ASNase), or vehicle control (control) and treated for an additional 14 days (**Figure 6A**). Tumors arising from liver cancer cells in the control mice grew steadily, but mice treated with Len had slower tumor growth. Mice treated with the Len with ASNase combination therapy exhibited completely retarded tumor growth (**Figure 6B**). Although Len or ASNase monotherapy suppressed tumor growth, the Len plus ASNase combination therapy was more effective than either of the monotherapies alone.

To assess the proliferation status of xenograft tumors, we performed Ki67 immunostaining. Consistent with tumor development, the Len alone and Len plus ASNase combination treatments had significantly reduced Ki67-positive tumor cell counts compared to ASNase monotherapy or the control condition (**Figure 6C**).

Although ASNase is associated with several toxicities, including liver dysfunction (25), we did not detect liver injury in the mice treated with ASNase. In turn, Len yielded mild liver injury, but the Len plus ASNase combination therapy did not exacerbate this injury (**Supplementary Figure 1C**). Further, Len treatment led to a mild increase in body weight relative to the control treatment, but this difference was not significant (**Supplementary Figure 1C**).

These findings suggest that ASNase sensitizes liver cancer cells to Len treatment, leading to synergistic inhibitory effects without exacerbation of liver injury.

## Discussion

Although glutamine is a nonessential amino acid, many cancer cells, including HCC cells, cannot survive without extracellular glutamine, and thus are thought to be dependent upon glutamine uptake (18). In both sarcoma cells (26) and HCC cells (9), GS, the rate-limiting enzyme required in glutamine synthesis, is needed to adapt to glutamine deprivation and for survival. High levels of GS expression are required for survival of cancer cells when extracellular glutamine is depleted. Importantly, as extracellular glutamine levels decline exogenous asparagine becomes an essential amino acid that can maintain protein synthesis and rescue growth and survival of glutamine-deprived tumor cells (6, 27). This pro-survival effect of asparagine has been confirmed for a variety of cell lines (27). The asparagine-mediated rescue of tumor cell proliferation is correlated with global protein synthesis, which requires GS (28, 29). In fact, proliferation was compromised in GS-deleted cells under conditions of glutamine depletion and exogenous asparagine supplementation. Both glutamine and asparagine depletion can be an approach for development of novel anti-HCC therapies, especially for management of tumor cells that express low levels of GS.

In the present study, ASNase, an enzyme derived from *Escherichia coli*, depleted glutamine and asparagine both in cell culture media and in the blood of mice (**Supplementary Figures 1A, B**). We examined the expression levels of GS and ASNS and explored the potential effects of ASNase on glutamine and asparagine production in several HCC cell lines. Although most HCC cells have high GS expression, a subset has downregulated GS expression that is similar to several ovarian cancer cell lines (30). These two HCC cell lines, SNU387 and SNU398, exhibited high ASNS and relatively low GS expression levels. ASNase treatment could inhibit the proliferation of HCC cells expressing low levels of GS. Interestingly, ASNase induces GS expression to a greater extent in HCC cells that had high GS expression levels (i.e., Hep3B, Huh7, Huh6, and HepG2 cells) (**Figure 4C**). This induction could contribute to ASNase resistance *via* acceleration of glutamine synthesis. Meanwhile, ASNS expression levels were inversely correlated with that of GS in the HCC cells analyzed in this study.

Even though glutamine-depleted cells have upregulated ASNS levels (31), this enzyme further reduces glutamine levels because ASNS catabolizes glutamine to glutamate. Notably, SNU387 and SNU398 cells, which exhibited markedly low GS and high ASNS expression levels, were more sensitive to ASNase than other HCC cell lines. The expression levels of GS and ASNS, which appear to be redundant, may predict the efficacy of ASNase therapy.

*GLUL* encodes GS that is also involved in the glutamine dependence of tumor cells and is a transcriptional target of β-catenin (15). Notably, activating β-catenin mutations frequently occur in liver tumors. As such, ASNase would be expected to inhibit the survival and proliferation of liver tumors with β-catenin mutations. Although we observed no positive correlation between the presence of β-catenin mutations (Huh6, SNU398, and HepG2 cells) and the efficacy of ASNase (**Figures 1A, B**), the expression levels of GS and ASNS seemed to have a potential impact on the effectiveness of ASNase treatment.

The mTORC1 pathway is a major oncogenic pathway in HCC (32). We and others have reported that hyperactivation of mTORC1 is sufficient to induce HCC in a mouse model (21, 22). Considering a previous report showing that GS induction of mTORC1 activation leads to tumor development (18), we assessed mTORC1 activity in HCC cells treated with Len or ASNase alone or Len with ASNase in combination and found heterogeneous mTORC1 activity among the tested cell lines (**Figures 4B, C**). We also reported that only aberrantly hyperactivated mTORC1 is a suitable target for HCC treatment (33). Since none of the treatments in this study yielded significant mTORC1 activation or suppression in Huh6 cells, mTORC1 may not be a critical determinant of the sensitivity to ASNase, at least in combination with Len.

Finally, we evaluated apoptotic cell death using poly (ADP-ribose) polymerase (PARP) as an apoptotic marker (**Figure 4C**). ASNase did not regulate PARP levels either with or without Len co-administration. Instead, ASNase induced ROS production and the Len + ASNase combination treatment enhanced this induction (**Figures 5A, B**). For glutamine-dependent cancers, drugs that disrupt intracellular redox homeostasis exert strong antitumor effects mediated through ROS production (24).

Collectively, these results suggest that glutamine and asparagine are critical for cancer cell survival, and depletion of these amino acids induces cellular oxidative stress, although further investigations are needed to confirm this hypothesis.

Len is one of the most effective currently available anti-HCC drugs (2); thus, novel combination strategies involving this drug are of interest. In fact, immuno-oncology drugs, such as immune checkpoint inhibitors, have been considered recently as potential partner drugs for TKIs like Len (34). Previous studies showed that the level of FGF19 expression is correlated with the efficacy of Len (35). Here we showed, as expected, that Hep3B and Huh7 cells, which have FGF19 mutation and activation of FGF signaling, showed increased sensitivity to Len relative to other cells (**Figures 1A, B**). Surprisingly, the combination of Len plus ASNase demonstrated synergistic effects in most cells lines with FGF19 mutations, regardless of Len or ASNase monotherapy treatment efficacy (**Figures 2A, B**). However, Huh7 cells were resistant to the effects of ASNase, possibly because these cells lack ASCT2, a critical transporter that uses extracellular glutamine in tumor cells (20). Huh7 cells may instead depend upon nutrients other than extracellular glutamine or on *de novo* glutamine synthesis.

In summary, here we showed that targeting amino acids that are most important for survival of liver cancer cells, glutamine and asparagine, *via* a combination therapy including the TKI Len and ASNase suppressed liver cancer cell proliferation *in vitro* and *in vivo*. To the best of our knowledge, this is the first report to address the effects of combination therapy of a TKI and a drug targeting amino acids. ASNase monotherapy was only effective in a subset of liver cancer cells having low GS and high ASNS expression, whereas when combined with Len a synergistic inhibitory effect on cell proliferation and tumor development was seen. Although the precise mechanism by which ASNase with and without Len co-administration suppresses liver cancer cells requires further investigation, our findings provide a basis for the development of a new therapeutic approach targeting amino acids *via* the use of drugs such as ASNase in combination with Len, which is now frequently used as a primary first-line TKI for HCC treatment.

### Limitations

The precise mechanism by which Len and ASNase combination therapy is synergistically effective—even in cells that are resistant to each drug on its own—remains unclear. ASNase monotherapy was not effective in Huh6 cells *in vitro*; however, it suppressed tumor development in the xenograft mouse model. Len was also more effective *in vivo* vs. in *in vitro* settings. This discrepancy may be attributed to the effects of ASNase on the tumor microenvironment, including the blood supply, immune cells, and other mesenchymal cells.

## Data Availability Statement

The original contributions presented in the study are included in the article/**Supplementary Material**. Further inquiries can be directed to the corresponding author.

## Ethics Statement

The animal study was performed in accordance with the guidelines of the care and use of live animals of the Kyoto Prefectural University of Medicine (KPUM) and was approved by the KPUM Institutional Animal Care and Use Committee (M2019-559).

## Author Contributions

KO: conceptualization, analysis, writing-original draft, and acquisition of data. AU: conceptualization, project administration, acquisition and analysis of data, funding acquisition, and writing-original draft. SK: conceptualization and study design. KoY: conceptualization and study design. AT: conceptualization and study design. SO: conceptualization and study design. HT: conceptualization and study design. YS: supervision and interpretation of data. TN: supervision and interpretation of data. KaY: supervision and interpretation of data. MM: supervision and interpretation of data. HN: conceptualization, interpretation of data, funding acquisition, refined important points of the manuscript. YL: supervision and interpretation of data. YM: resources, conceptualization and study design. YK: resources, conceptualization and study design. TS: resources, conceptualization and study design. TO: resources, conceptualization, study design, funding acquisition and supervision. YI: conceptualization, study design, interpretation of data, funding acquisition, supervision and writing–original draft. All authors: drafting and/or revising this work. All authors contributed to the article and approved the submitted version.

## Funding

This research was supported by governmental grants from the Japan Society for the Promotion of Science, Grants-in-Aid for Scientific Research (KAKENHI) #19K08377 (AU), AMED #JP19fk0210059 (AU and HN), #JP18fk0210027 (KY and YI), #JP19fk0210040 (TO), #JP18fk0210040 (YI), MEXT Grant-in-Aid for Scientific Research (S) #16H06389 (TO).

## Conflict of Interest

AU received commercial research funding from AbbVie, Inc., and Merck Sharp & Dohme Corp. MM received lecture fees from Eisai Co., Ltd. YI received lecture fees from Merck Sharp and Dohme, and Eisai Co., Ltd. as well as commercial research funding from Bayer AG, Eisai Co., Ltd., Merck Sharp and Dohme, Takeda Pharmaceutical Company, Limited, and Chugai Pharmaceutical Co., Ltd.

The remaining authors declare that the research was conducted in the absence of any commercial or financial relationships that could be construed as a potential conflict of interest.

## Publisher’s Note

All claims expressed in this article are solely those of the authors and do not necessarily represent those of their affiliated organizations, or those of the publisher, the editors and the reviewers. Any product that may be evaluated in this article, or claim that may be made by its manufacturer, is not guaranteed or endorsed by the publisher.
