# Supplementary material for: Enhanced Antitumor Effect in Liver Cancer by Amino Acid Depletion-Induced Oxidative Stress
Source: Front Oncol. 2021 Nov 2;11:758549. doi: 10.3389/fonc.2021.758549 (PMC8593418; doi:10.3389/fonc.2021.758549)
Supplement: Supplementary file 1 [file DataSheet_1.docx]

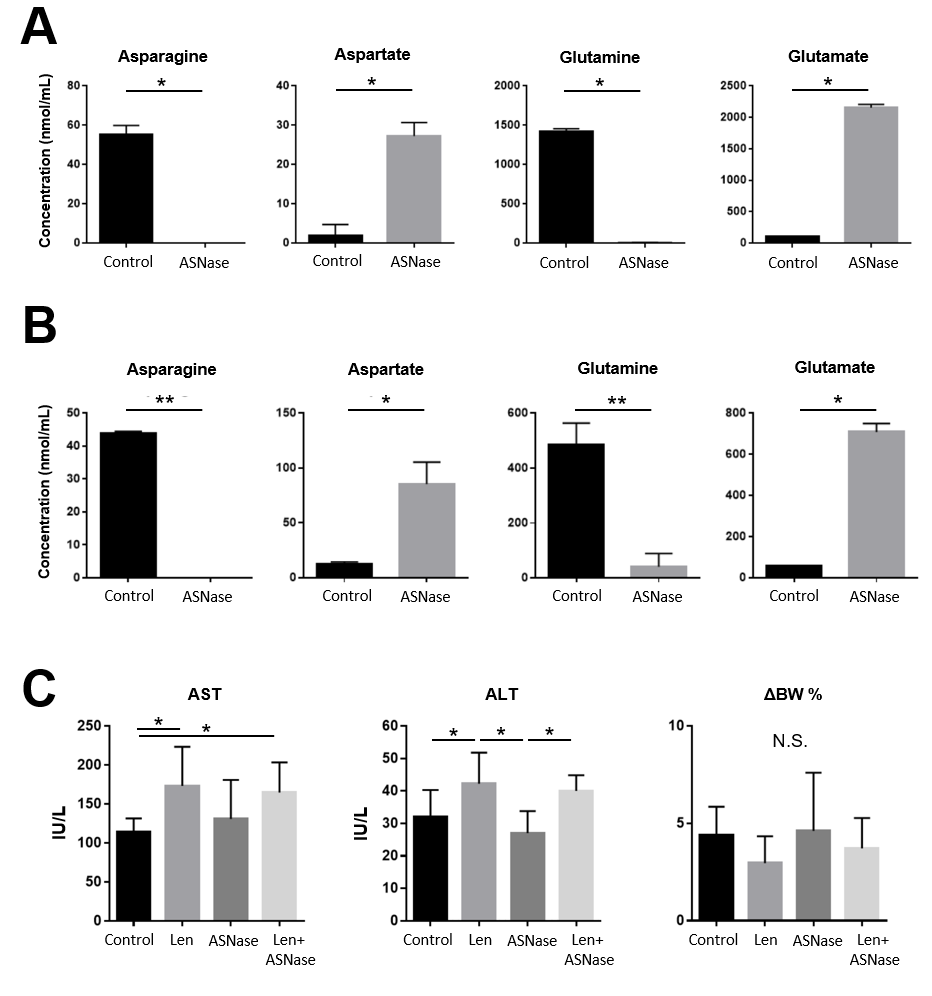
**Figure S1.** **Effects of l-asparaginase on cultured cells and mice**

(A) Amino acid concentrations in cell culture media. Huh6 cells were incubated in culture medium containing 3.13 U/ml ASNase for 24 h. Culture media were collected and analyzed by Liquid Chromatography-Mass Spectrometry (LC/MS) (n = 3 per group, student’s t-test). (B) Amino acid concentrations in the blood of mice. Wild-type C57/B6J mice were intraperitoneally injected with ASNase at 3 U/g body weight once daily for 8 consecutive days. PBS was injected in the control group (n = 4, control group; n = 8, ASNase group, student’s t-test). (C) Serum levels of AST and ALT and body weight (BW) gain (%) were analyzed in the xenografted mice upon sacrifice. (Lenvatinib (Len), l-asparaginase (ASNase), Lenvatinib and l-asparaginase combination (Len + ASNase), or vehicle control (Control)) (Turkey’s multiple comparisons test, not significant (N.S.), **P* < 0.05, ***P* < 0.01).

**Figure S2. Effects of single or combination treatment against normal mouse hepatocytes**

**
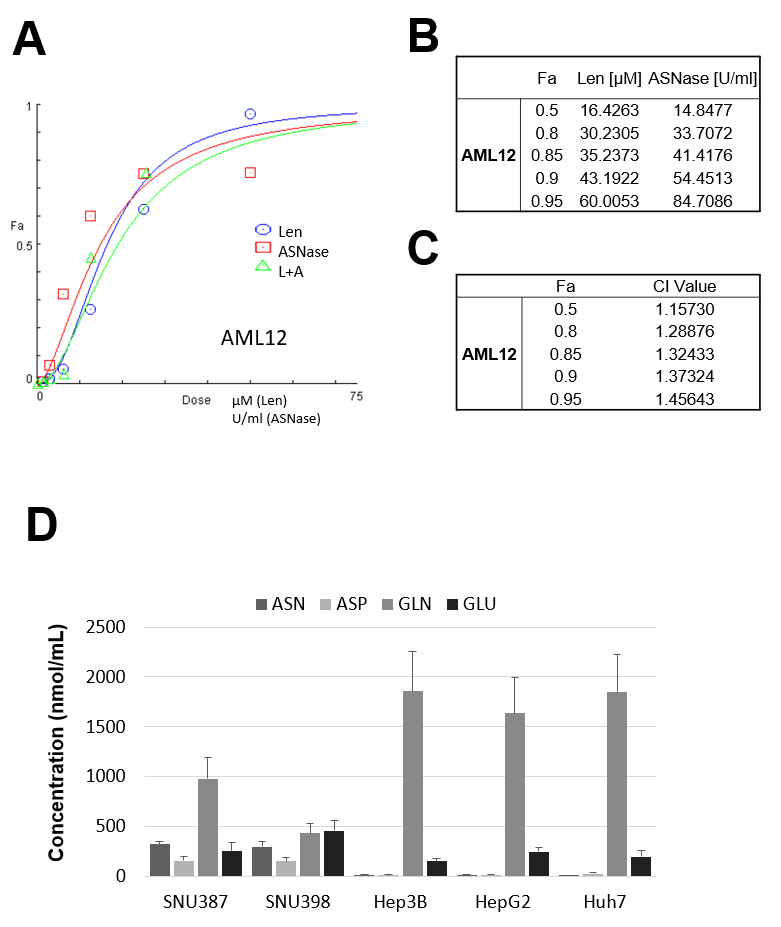
**

Effects of Lenvatinib (Len), l-asparaginase (ASNase) or Len + ASNase combination treatment on AML12 cells. AM12 cells were incubated with indicated concentrations of the drugs for 72 hours. (A) Dose-effect curves were generated using cell viability data from WST-8 assays. (B) The fraction of affected cells (Fa) representing the percentage of growth inhibition and dose-response plots are indicated for monotherapy and combination therapy (Len, blue; ASNase, red; Len plus ASNase, green). Drug doses that yield Fa 0.5 are required for a 50% inhibitory effect (equivalent to the IC50). (C) Summary of drug concentrations that inhibited cell survival by 50%, 80%, 85%, 90%, and 95%, indicated by Fa. Representative data from three independent experiments were shown. (D) Amino acid concentrations in culture media from the indicated cell lines. (Asparagine (ASN), aspartate (ASP), glutamine (GLN), glutamate (GLU)).

**Figure S3. Effects of GS and ASNS knockdown on HCC cell proliferation**

**
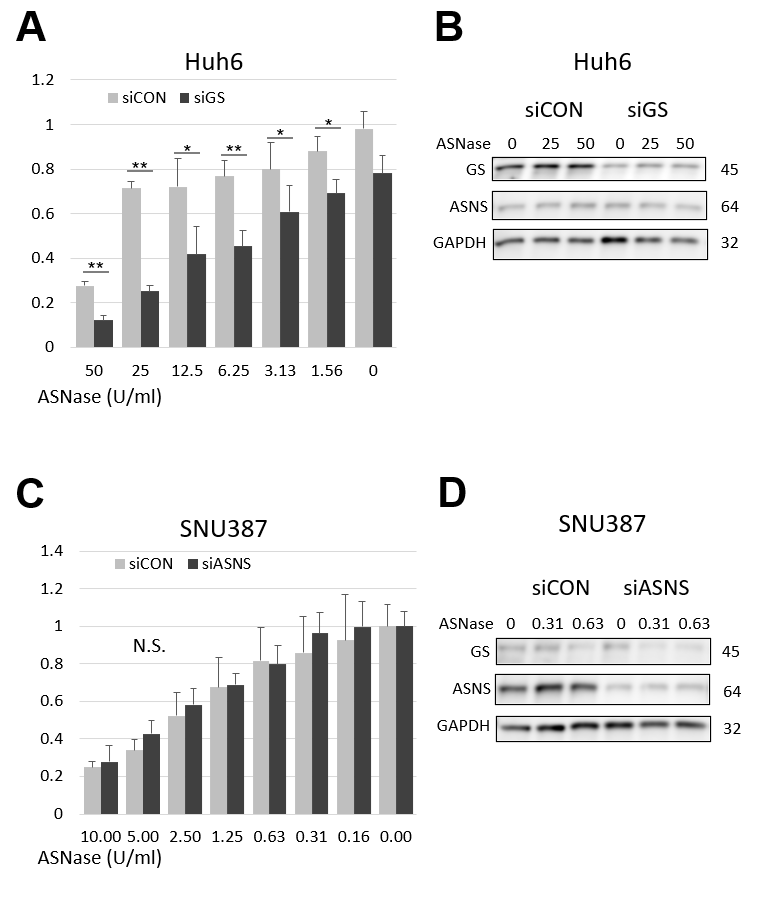
**

(A) Cell viability/proliferation of Huh6 cells with siRNA-mediated GS knockdown incubated with the indicated amount of ASNase. (B) Protein expression of GS and ASNS in HCC cells treated with the indicated amount of ASNase (U/ml) (siCON, nonspecific negative control siRNA; siGS, GS siRNA). (C) Cell viability/proliferation of SNU387 cells with siRNA-mediated ASNS knockdown incubated with the indicated amount of ASNase. (D) Protein expression of GS and ASNS in HCC cells treated with the indicated amount of ASNase (U/ml) (siASNS, ASNS siRNA). All graphs show the mean ± SD, and representative data from three independent experiments were shown (not significant (N.S.), *P < 0.05, **P < 0.01).

**Figure S4. Effects of GS and ASNS knockdown on HCC cell proliferation**

**
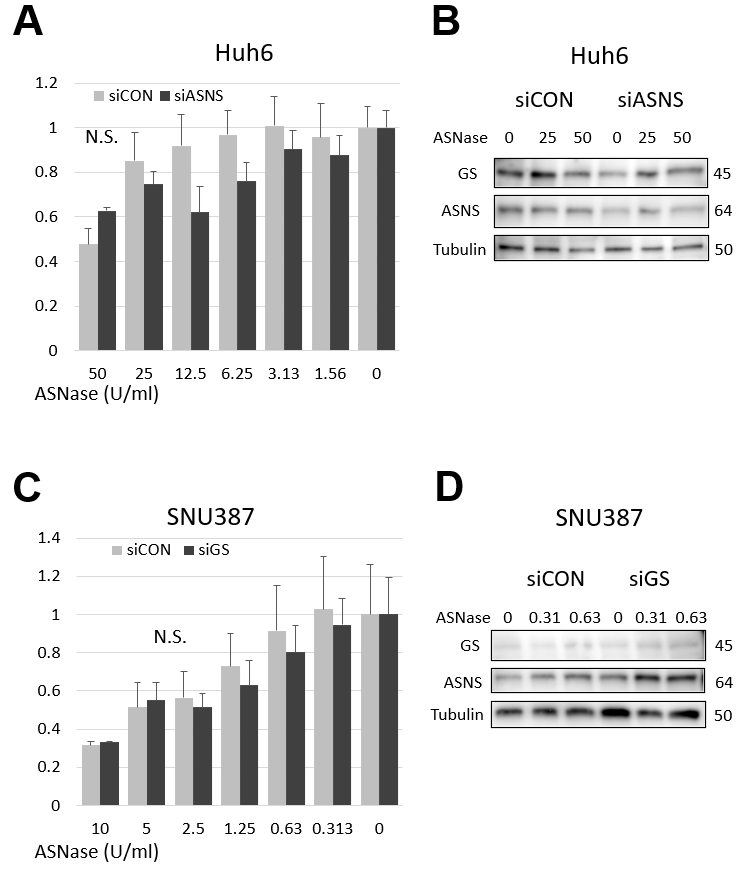
**

(A) Cell viability/proliferation of Huh6 cells with siRNA-mediated ASNS knockdown incubated with the indicated amount of ASNase. (B) Protein expression of GS and ASNS in HCC cells treated with the indicated amount of ASNase (U/ml) (siCON, nonspecific negative control siRNA; siASNS, ASNS siRNA). (C) Cell viability/proliferation of SNU387 cells with siRNA-mediated GS knockdown incubated with the indicated amount of ASNase. (D) Protein expression of GS and ASNS in HCC cells treated with the indicated amount of ASNase (U/ml) (siGS, GS siRNA). All graphs show the mean ± SD and representative data from three independent experiments were shown (not significant (N.S.)).

**Table S1. Primers used in this study**

| *GLUL*_F 5′- TCATCTTGCATCGTGTGTGTG - 3′ |
| --- |
| *GLUL*_R 5′- CTTCAGACCATTCTCCTCCGG - 3′ |
| *ASNS*_F 5′- GATTGCCTTCTGTTCAGTGTCT - 3′ |
| *ASNS*_R 5′- GGGTCAACTACCGCCAACC - 3′ |
| *ASCT2*_F 5′- TGGTCTCCTGGATCATGTGG - 3′ |
| *ASCT2*_R 5′- TTTGCGGGTGAAGAGGAAGT - 3′ |
| *GAPDH*_F 5′- TGACAACTTTGGTATCGTGGAAGG - 3′ |
| *GAPDH*_R 5′- AGGCAGGGATGATGTTCTGGAGAG - 3′ |

**Supplementary Materials and Methods**

**siRNA knockdown**

Small-interfering RNAs (siRNAs) were obtained from Thermo Fisher Scientific. Huh6 and SNU387 cells were transfected with GS (#s1679) and ASNS siRNA (#s421). Nonspecific siRNA (#4390843) was used as a negative control. Lipofectamine-RNAi max and Opti-MEM I were purchased from Life Technologies.
